# Supplementary material for: Scalable production and purification of engineered ARRDC1-mediated microvesicles in a HEK293 suspension cell system
Source: Sci Rep. 2025 Mar 1;15:7299. doi: 10.1038/s41598-025-87674-5 (PMC11873033; doi:10.1038/s41598-025-87674-5)
Supplement: Supplementary file 1 — Supplementary Information 1. [file 41598_2025_87674_MOESM1_ESM.docx]

Supplementary Figure Legends

Supplemental Figure 1. Distribution of particles and GFP after UC. Particle concentrations were determined by NTA, and GFP concentrations were determined by ELISA. The total amount (concentration x volume) of each is shown in the CM, supernatant, and re-suspended pellet.

Supplemental Figure 2. GFP co-localization with tetraspanins. Particles from the control and transfected samples were labeled with antibodies to **(A)** CD9, **(B)** CD63, **(C)** CD81, and **(D)** a mix of these, and analyzed nano-flow cytometry. For each chart, the red bar indicates the percentage of events that were labeled with the antibody, and the green bar represents the percentage of GFP+ events. The yellow bar indicates the events that were positive for both GFP and the antibody label.

Supplemental Figure 3. Figure 1D uncropped western blots and raw images used for pixel densitometry analysis.

Supplemental Figure 4. Figure 2D and 2E uncropped western blots and raw images used for pixel densitometry analysis.

Supplemental Figure 5. Figure 4B uncropped western blots and raw images used for pixel densitometry analysis.

Supplemental Figure 6. Figure 5G uncropped western blots and raw images used for pixel densitometry analysis.

Supplemental Figure 7. Figure 5H uncropped western blots and raw images used for pixel densitometry analysis.

Supplemental Figure 8. Figure 8 uncropped western blots and raw images used for pixel densitometry analysis.
