# Supplementary material for: Scalable production and purification of engineered ARRDC1-mediated microvesicles in a HEK293 suspension cell system
Source: Sci Rep. 2025 Mar 1;15:7299. doi: 10.1038/s41598-025-87674-5 (PMC11873033; doi:10.1038/s41598-025-87674-5)

Supplemental Figure 1

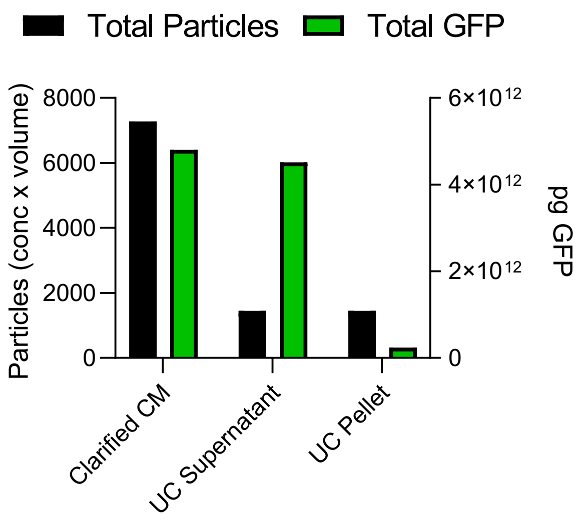

Supplemental Figure 2

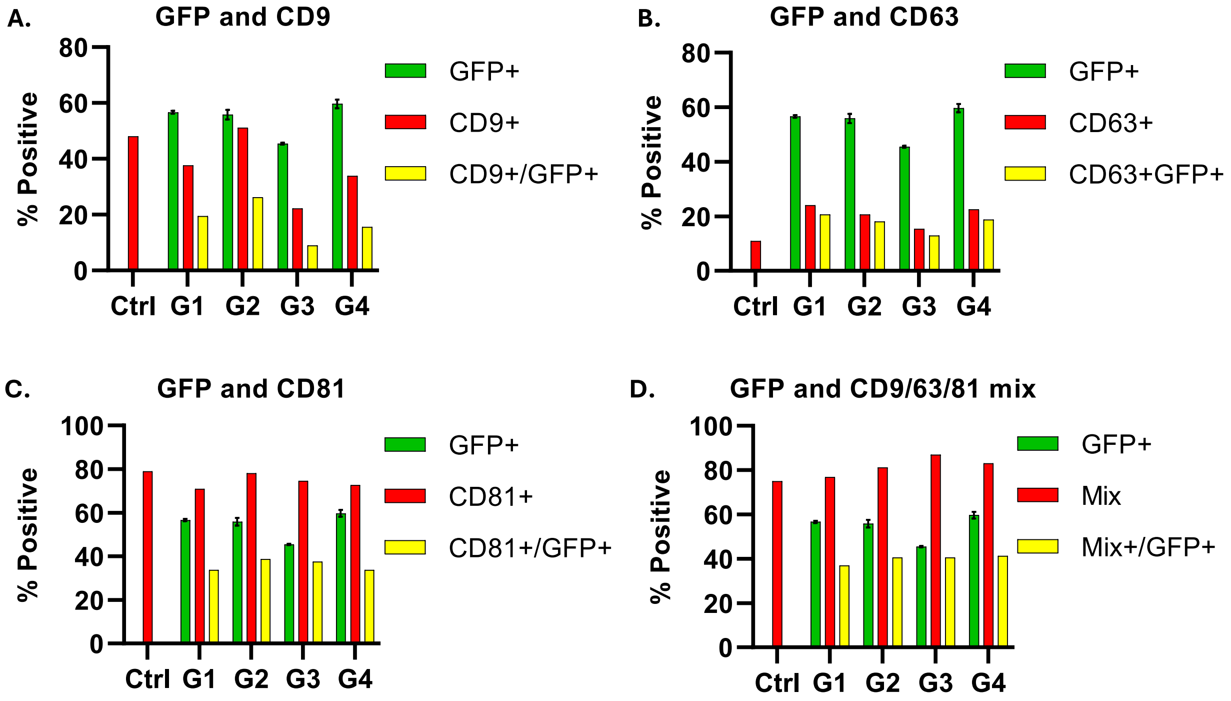

Supplemental Figure 3

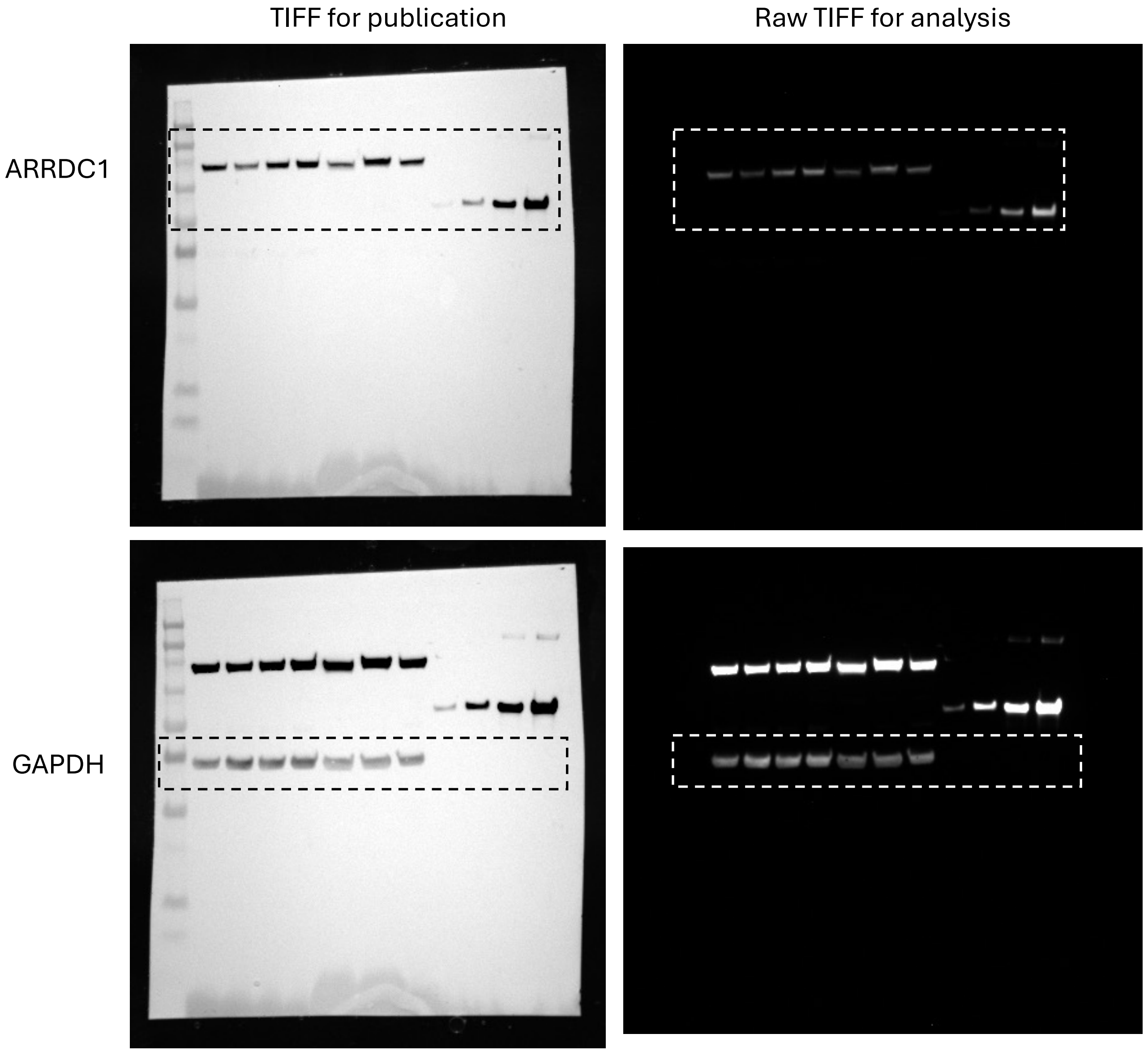

Supplemental Figure 4

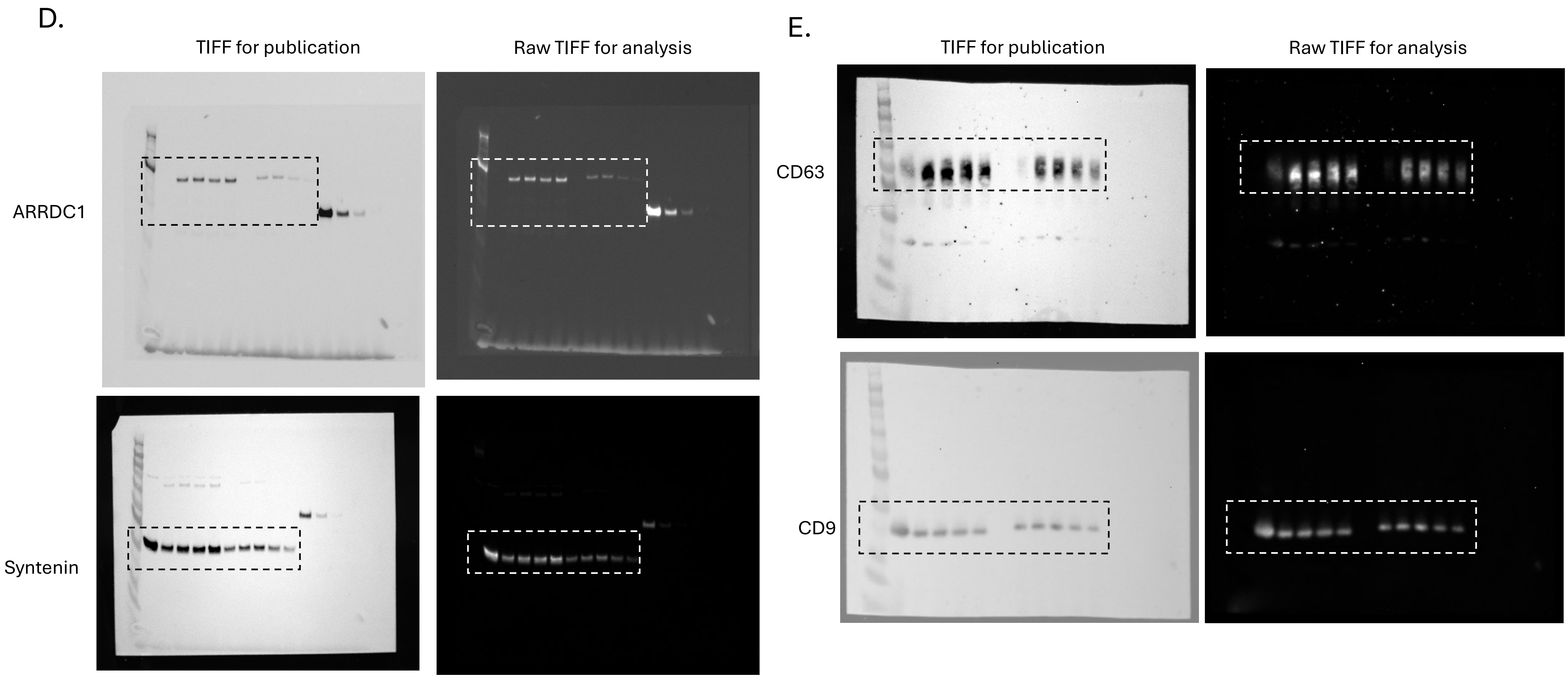

Supplemental Figure 5

ARRDC1

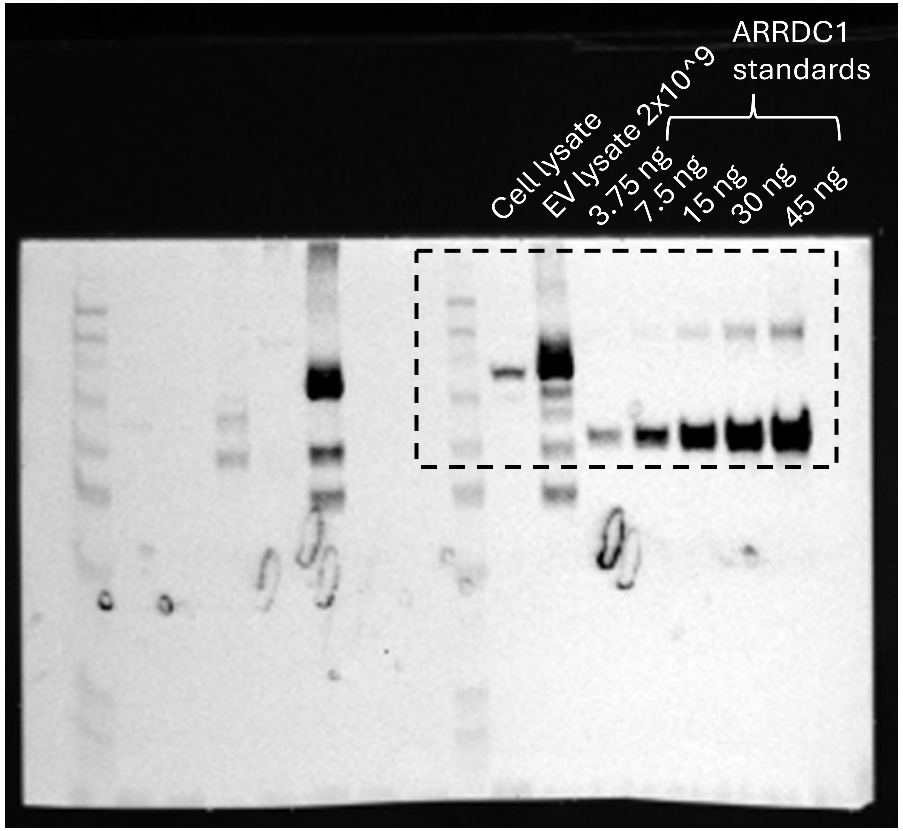

Supplemental Figure 6

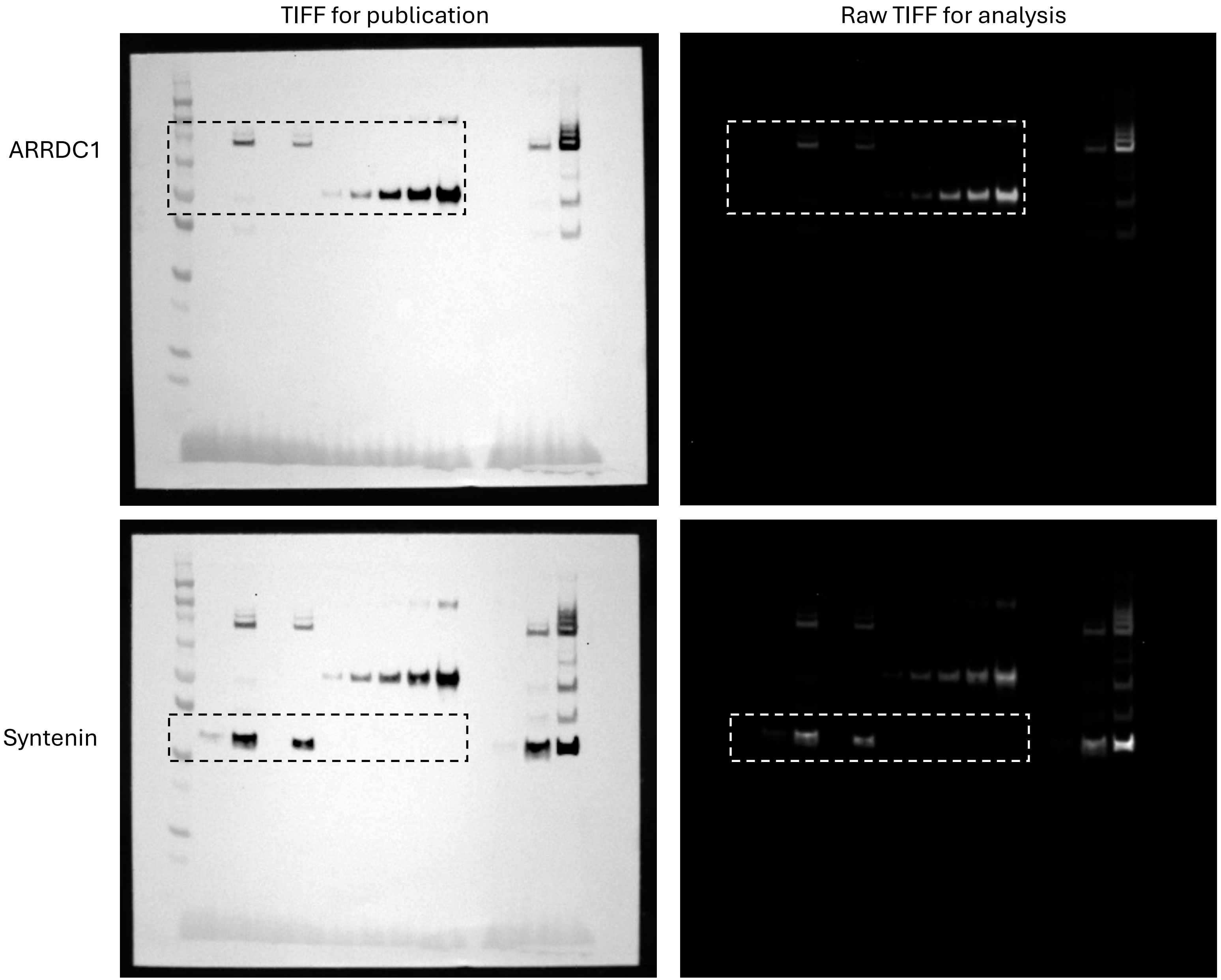

Supplemental Figure 7

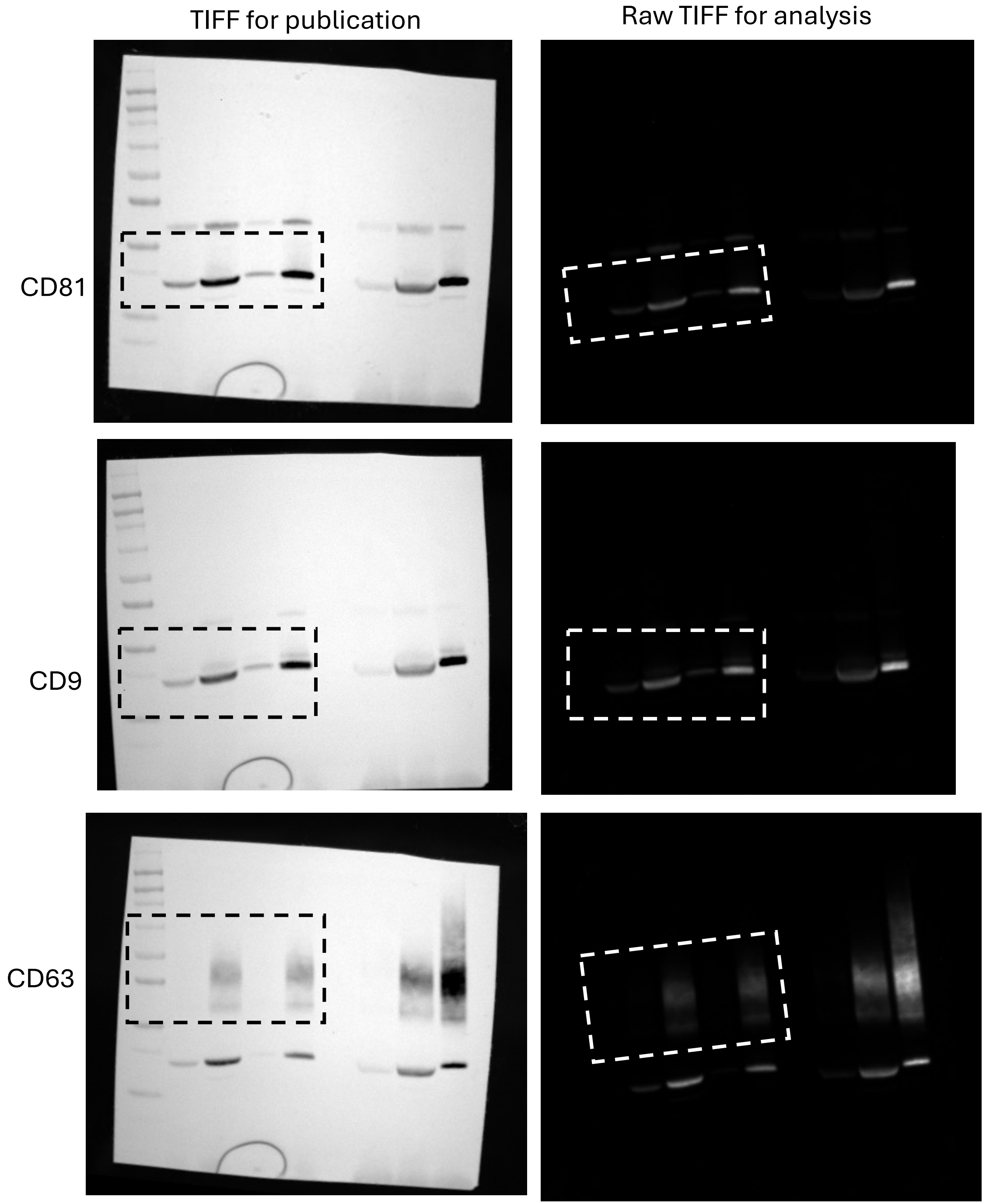

Supplemental Figure 8

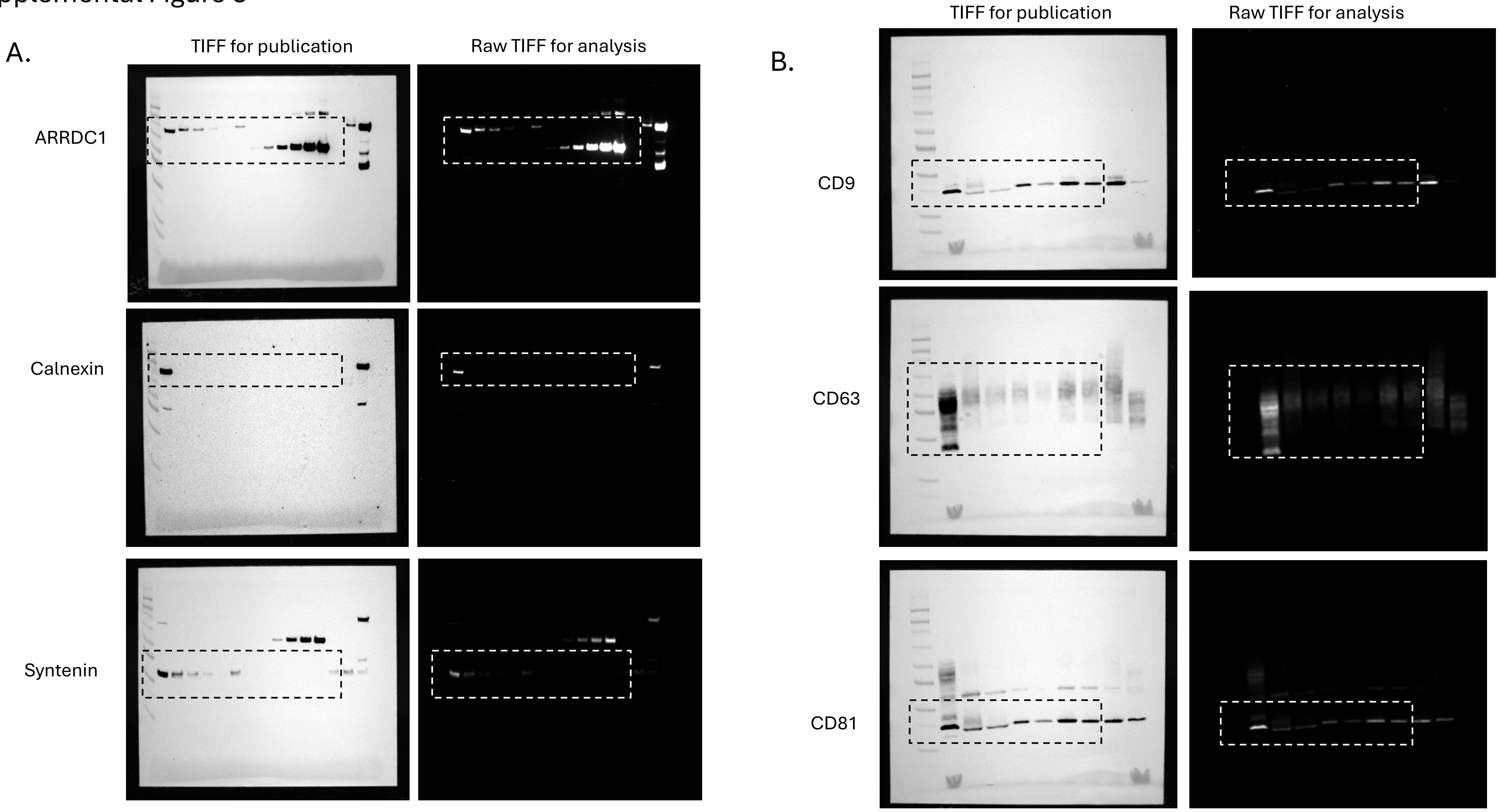

Supplement: Supplementary file 2 — Supplementary Information 2. [file 41598_2025_87674_MOESM2_ESM.pdf]
